# Sickle cell maculopathy: Identification of systemic risk factors, and microstructural analysis of individual retinal layers of the macula

**DOI:** 10.1371/journal.pone.0193582

**Published:** 2018-03-01

**Authors:** Laura Dell’Arti, Giulio Barteselli, Lorenzo Riva, Elisa Carini, Giovanna Graziadei, Eleonora Benatti, Alessandro Invernizzi, Maria D. Cappellini, Francesco Viola

**Affiliations:** 1 Department of Clinical Sciences and Community Health, University of Milan, Milan, Italy; 2 Ophthalmological Unit, Ca’ Granda Foundation, IRCCS Ospedale Maggiore Policlinico, Milan, Italy; 3 Genentech Inc, South San Francisco, California, United States of America; 4 Rare Diseases Center, Department of Medicine and Medical Specialties, Ca’ Granda Foundation, IRCCS Ospedale Maggiore Policlinico, Milan, Italy; 5 Eye Clinic, Department of Biomedical and Clinical Science "L. Sacco", Luigi Sacco Hospital, University of Milan, Milan, Italy; University of Florida, UNITED STATES

## Abstract

**Purpose:**

To identify systemic risk factors for sickle cell maculopathy, and to analyze the microstructure of the macula of Sickle Cell Disease (SCD) patients by using automated segmentation of individual retinal layers.

**Methods:**

Thirty consecutive patients with SCD and 30 matched controls underwent spectral-domain optical coherence tomography (SD-OCT) and automated thickness measurement for each retinal layer; thicknesses for SCD patients were then compared to normal controls. Demographic data, systemic data, and lab results were collected for each SCD patient; multivariate logistic regression analysis was used to identify potential risk factors for sickle cell maculopathy.

**Results:**

Ongoing chelation treatment (p = 0.0187) was the most predictive factor for the presence of sickle cell maculopathy; the odds were 94.2% lower when chelation was present. HbF level tended to influence sickle cell maculopathy (p = 0.0775); the odds decreased by 12.9% when HbF increased by 1%. Sickle cell maculopathy was detected in 43% of SCD patients as patchy areas of retinal thinning on SD-OCT thickness map, mostly located temporally to the macula, especially in eyes with more advanced forms of sickle cell retinopathy (p = 0.003). In comparison to controls, SCD patients had a subtle thinning of the overall macula and temporal retina compared to controls (most p<0.0001), involving inner and outer retinal layers. Thickening of the retinal pigment epithelium was also detected in SCD eyes (p<0.0001).

**Conclusions:**

Chronic chelation therapy and, potentially, high levels of HbF are possible protective factors for the presence of sickle cell maculopathy, especially for patients with more advanced forms of sickle cell retinopathy. A subtle thinning of the overall macula occurs in SCD patients and involves multiple retinal layers, suggesting that ischemic vasculopathy may happen in both superficial and deep capillary plexi. Thinning of the outer retinal layers suggests that an ischemic insult of the choriocapillaris may also occur in SCD patients.

## Introduction

Approximately 60,000 people in the USA and 10,000 in the United Kingdom suffer from Sickle Cell Disease (SCD), making it one of the most common genetic disorders.[1] In SCD, erythrocytes deform into a sickle shape, resulting in vaso-occlusive complications in multiple organs.

Ocular complications are commonly observed in SCD, and involve the retina in particular. Proliferative sickle cell retinopathy (PSR) is the major sight-threatening complication, most frequently observed in hemoglobin SC disease.[1] Goldberg in the early 70’s extensively studied PSR, and described 5 stages characterized by peripheral retinal ischemia, peripheral neovascularization, intravitreal hemorrhage, and tractional or mixed retinal detachment.[2] This peculiar retinopathy has been of particular interest in several studies as it is considered a model of purely ischemic retinopathy without an exudative component.[3]^-^[4] Another emerging complication is *‘sickle cell maculopathy’*, which is a localized macular thinning secondary to ischemia. Sickle cell maculopathy is a subtle disease which is not always clinically apparent on indirect ophthalmoscopy; instead, it is more easily detectable using spectral domain optical coherence tomography (SD-OCT) and OCT angiography (OCT-A). Early histopathologic studies demonstrated loss of the inner retinal layers in SCD patients[5], while isolated studies using SD-OCT have shown inner or outer macular thinning without clinical evidence of nonperfusion on fluorescein angiography (FA)[6–8], as well as patchy areas of severe retinal thinning in the temporal macula.[8, 9] The exact etiology of macular thinning in the absence of clinically apparent nonperfusion still remains unclear. However, recent OCT-A evidence has suggested that nonperfusion of the retinal capillary plexi may be responsible for the areas of macular thinning seen in SCD patients that was seen on SD-OCT.[9–12] As Martin et al have demonstrated[13], temporal macular atrophy in sickle cell disease may have direct consequences on visual function even when the visual acuity is preserved. Indeed, areas of retinal thinning in asymptomatic patients were found to match with paracentral scotomas on automated perimetry. Therefore, irreversible visual loss can occur either secondary to PSR or sickle cell maculopathy, and identifying patients at risk for SCD ocular complications can be crucial to limit drastic consequences for patients’ vision and quality of life. Previous investigators have demonstrated that PSR severity is potentially associated with SC genotype, lower fetal hemoglobin (HbF), and higher hemoglobin levels.[14] It has also been reported that circulating PEDF and low sICAM-1 are associated with sickle cell retinopathy.[4] On the contrary, given that sickle cell maculopathy has been studied only recently, demographic or systemic risk factors for the occurrence of irreversible severe retinal thinning in sickle cell maculopathy have not been evaluated yet.

The primary purpose of the present study was to identify the potential role of demographic and systemic factors not yet investigated on the occurrence of sickle cell maculopathy, in order to provide useful information to clinicians who are managing SCD patients from the hematological and ophthalmological standpoint. The secondary purpose was to analyze the microstructure of the macula of SCD patients by evaluating the thickness of each individual retinal layer using an automated software for retinal segmentation; such evaluation may bring further evidence and eventually provide a more comprehensive explanation of the occurrence and implications of sickle cell maculopathy.

## Materials and methods

### Patients

The research adhered to the tenets of the Declaration of Helsinki, and approval by the investigational review board of the Fondazione IRCCS Cà Granda, Ospedale Maggiore Policlinico, was obtained. A signed written informed consent form was obtained from each participant (SCD patients and controls). This prospective, cross-sectional, case-control study was performed at the University of Milan, Ospedale Maggiore Policlinico of Milan. Patients with SCD were recruited in a tertiary referral center in Milan (Department of Internal Medicine, Rare Diseases Center, Fondazione IRCCS Ca’ Granda-Ospedale Maggiore Policlinico, Italy) and were evaluated consecutively at the Department of Ophthalmology of the same institution between March 2014 and October 2015. Only patients with age 18 or above and with electrophoretic confirmation of SCD (HbSS, HbSC, or HbS/β°) were recruited. Ophthalmic examinations were conducted after explanation of the procedures and receipt of written informed consent, and included best corrected visual acuity (BCVA) measurement, slit-lamp evaluation, indirect ophthalmoscopy, SD-OCT and FA. All examinations for an individual study participant were performed on the same day. If signs of retinal abnormalities not related to SCD were detected (e.g., inherited retinopathy, age-related macular degeneration, presence of epiretinal membrane, or diabetic retinopathy), patients were excluded from the study. In addition, we excluded patients with high myopia (> -6 diopters), with media opacities not allowing good-quality ocular imaging, and patients previously treated with panretinal photocoagulation for PSR. Data from both eyes of each participant were used for the analyses, unless there were ocular abnormalities that could affect visual function in either eye. An age and sex-matched control group of healthy patients was also recruited, and underwent the same ocular procedures, except for FA.

### Ocular imaging

#### 1) Fluorescein angiography

- Conventional FA was performed with series of early frames both with standard foveal fixation and temporal fixation (12.5 degrees temporally to the fovea), acquired with the 30-degree standard lens. Retinal periphery was studied evaluating the 9 standard fields (temporal-superior, superior, nasal-superior, nasal, inferior nasal, inferior, temporal-inferior, temporal and posterior pole) with the 55-degree lens or with Staurenghi lens (Staurenghi 230 SLO Retina Lens; Ocular Instruments Inc., Bellevue, Washington) at the physician’s discretion.
- Evaluation of macular vascular abnormalities and determination of the stage of sickle cell retinopathy were based on FA, and was performed by two trained independent physicians. In case of disagreement, a third physician was consulted to achieve an acceptable result. The diagnosis of macular vascular abnormalities included the presence of microaneurysms, ischemic areas, anastomosis, and/or hairpin-shaped venous loops.
- PSR features were graded according to the Goldberg classification[2] as follows: stage 0, fully vascularized peripheral retina; stage I, peripheral retinal ischemia; stage II, peripheral arteriovenous anastomosis; stage III, peripheral neovascularization; stage IV, intravitreous hemorrhage; stage V, retinal detachment. In case a patient presented with previously performed focal laser treatment for peripheral neovascularization, patients were classified as stage III. The severity of sickle cell retinopathy was obtained by classifying eyes into three groups according to the presence of proliferative changes: no retinopathy (stage 0), non proliferative retinopathy (i.e., Goldberg stage I and II), and proliferative retinopathy (i.e., Goldberg stage III, IV and V).

#### 2) Optical coherence tomography

**Fig 1:**
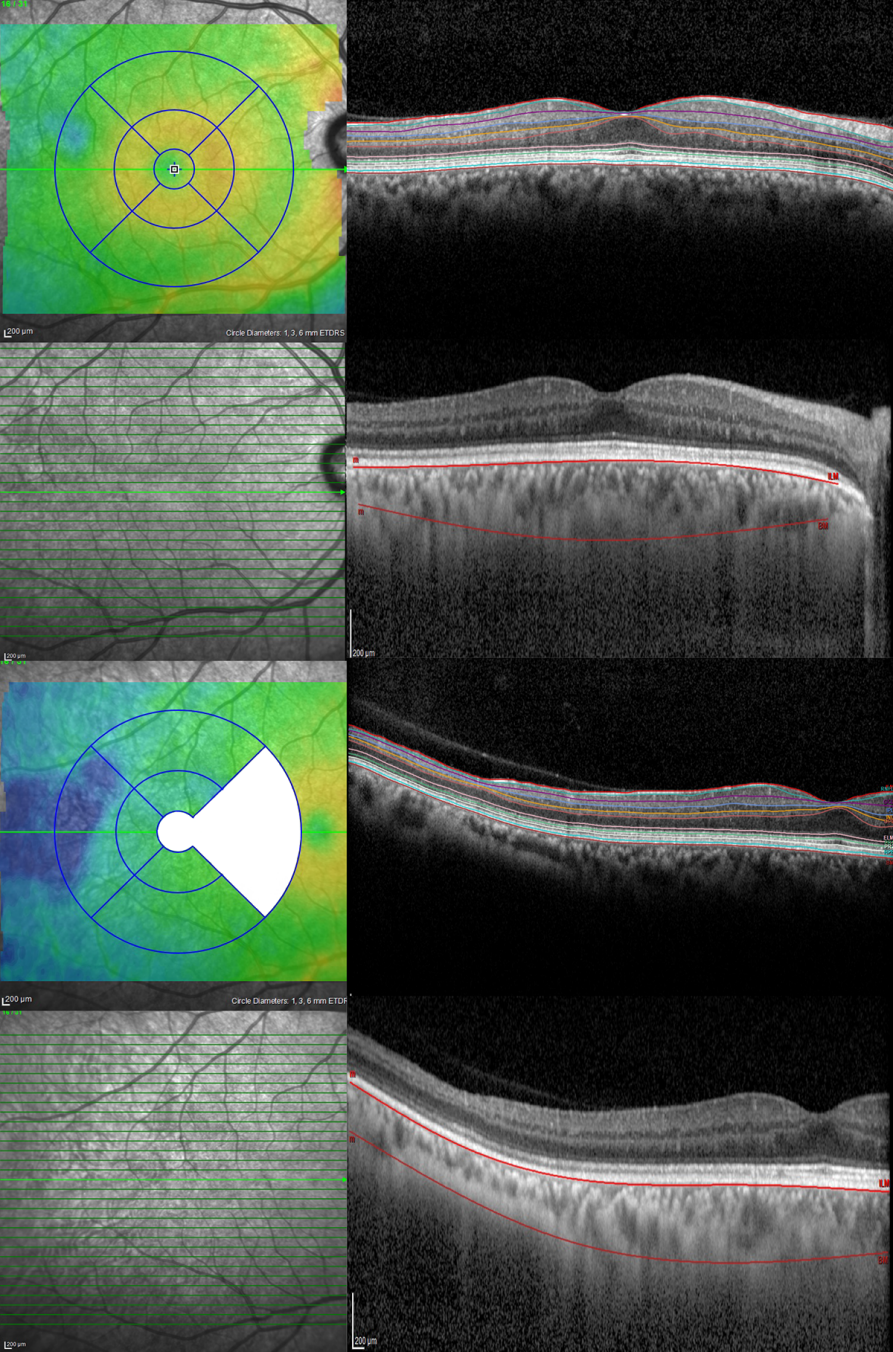
Optical coherence tomography imaging protocol and analysis, for both foveal-centered and temporally centered scans. (Left column) Color-coded retinal thickness maps were generated by the built-in software of the device, which then automatically applied the ETDRS grid after scanning eyes using 31 high-resolution B-scans. The nasal quadrant and the central ring of the temporally-centered scan were systematically not measured because overlapping with the temporal quadrant of the foveal-centered scan. (Right column) The segmentation software of the SD-OCT device automatically detected each retinal layer, including retinal nerve fiber layer, ganglion cell layer, inner plexiform layer, inner nuclear layer, outer plexiform layer, outer nuclear layer, photoreceptor layer, and retinal pigment epithelium. Segmentation of the choroid was performed manually.

**Fig 2:**
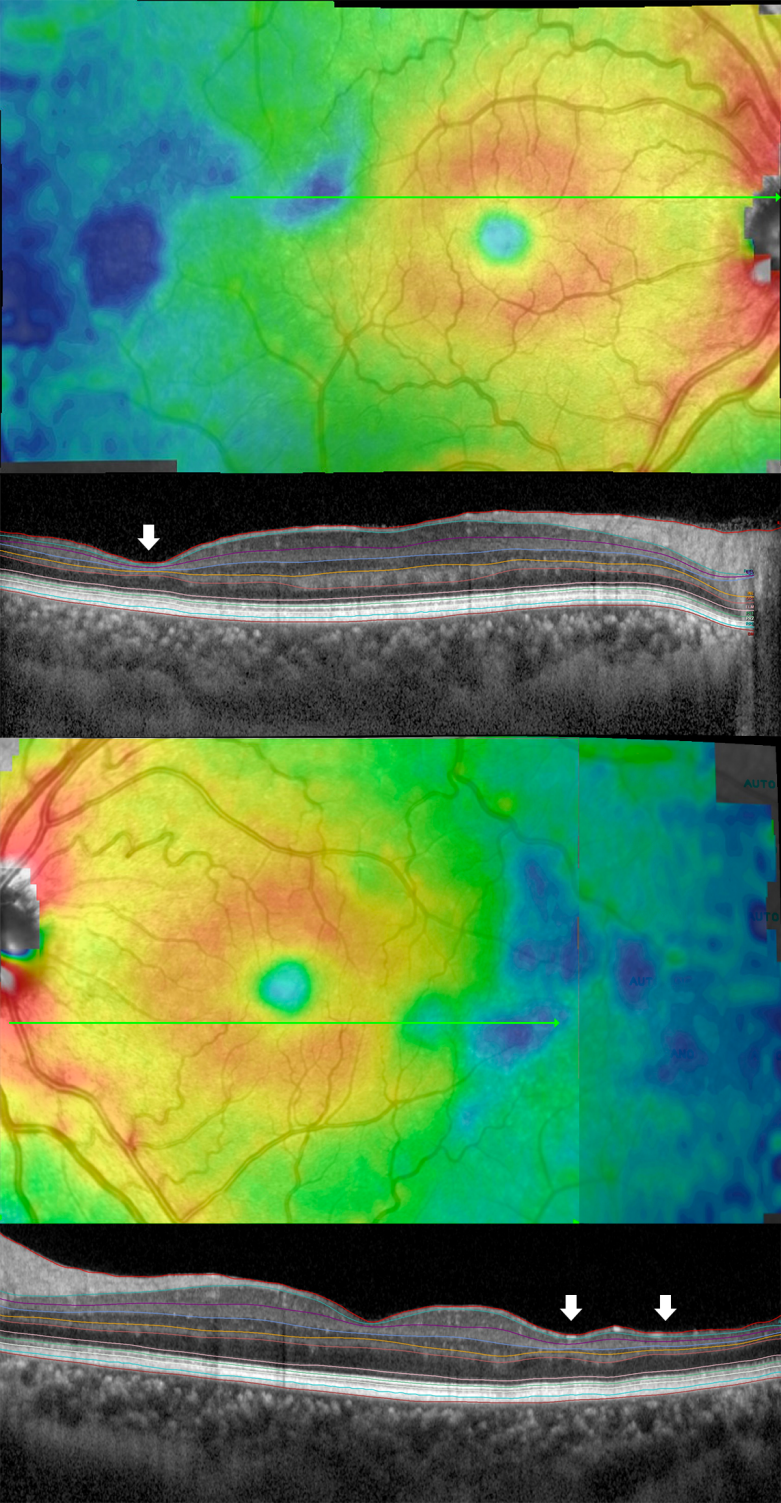
Example of sickle cell maculopathy. (First and third rows) Patchy areas of markedly reduced retinal thickness are visible as blue areas on retinal thickness maps. (Second and fourth row) Automatic segmentation of the B-scans passing through thinned areas demonstrates thinning of multiple individual retinal layers (white arrows).

- OCT was carried out using the Spectralis SD-OCT (Heidelberg Engineering, Heidelberg, Germany) for all patients; the standard scanning protocol included 31 high-resolution B-scans, and each scan was approximately 9 mm in length and spaced 240 microns apart. All 31 B-scans were acquired in a continuous, automated sequence and covered a 30° x 25° area. A minimum of 20 frames were averaged automatically and used to obtain a good quality image. The central fixation target was used to center the raster scan to the fovea while the 12.5-degree temporal fixation target was used to center the scan temporally to the fovea. The same protocol was repeated using the Enhanced Depth Imaging (EDI) mode to better study the choroid. (Fig 1).
- Color-coded retinal thickness maps centered on the fovea and temporally to the fovea were automatically generated by the built-in software of the device after applying the ETDRS grid (Fig 1). Measurements of retinal thickness and choroidal volume were recorded. The ETDRS grid divides the macula into 3 concentric rings (center, inner, and outer), with the inner ring measuring 1 to 3 mm and the outer ring measuring 3 to 6 mm of diameter (referring to a ring with a diameter of 1 mm). The grid further divides inner and outer rings into 4 quadrants (superior, inferior, temporal, and nasal). Thickness data from the nasal quadrant and central ring of the temporally-centered grid were not collected because of overlapping with the temporal quadrant of the fovea-centered grid (Fig 1).
- To study the choroid, one trained physician manually performed the choroidal segmentation using a previously described well-reproducible method[15], and then the software automatically provided the choroidal volume within the ETDRS grid (Fig 1).
- Individual retinal layers were automatically segmented using the built-in segmentation software of the Heidelberg Eye Explorer software, after which a trained ophthalmologist (GB) reviewed the results and occasionally corrected the segmentation lines where necessary. The segmentation software then provided thickness maps and measurements from each retinal layer in the ETDRS grid as follows; retinal nerve fiber layer (RNFL), ganglion cell layer (GCL), inner plexiform layer (IPL), inner nuclear layer (INL), outer plexiform layer (OPL), outer nuclear layer (ONL), photoreceptor layer (PR), and retinal pigment epithelium (RPE) thicknesses (Fig 1). A previous study has demonstrated that thickness estimates using the Heidelberg Eye Explorer software were highly repeatable for all retinal layers, except the OPL.[16]
- SCD eyes were divided into two groups based on the presence of sickle cell maculopathy, identified as patchy areas of severe retinal thinning on OCT (i.e., blue areas of markedly decreased thickness on retinal thickness color-coded map) (Fig 2).

### Data collection

Clinical data were collected from the patient’s hematological charts, and included demographics (age, gender, ethnicity), ocular history, medical history (SCD type, transfusion treatment, iron-chelation and hydroxyurea therapy, spleen status, previous ischemic events), hematology values (fetal hemoglobin (HbF), sickle hemoglobin (HbS), reticulocytes, leucocytes, neutrophils, hemoglobin, hematocrit, lactate dehydrogenase (LDH), total and indirect bilirubin, aspartate aminotransferase (AST), alanine aminotransferase (ALT), ferritin, transferrin saturation, mean corpuscular volume (MCV), platelets, creatinine, urea, uric acid). Labs value were recorded from the last on-site consultation, that was performed the same month of the ophthalmological evaluation.

### Statistical methods

Sickle Cell Disease patients were evaluated for potential risk factors which may influence sickle cell maculopathy (occurring in one or both eyes). Univariate logistic regression was used to model the proportion of patients with sickle cell maculopathy for multiple demographic and systemic factors. A multivariate logistic regression model was then fit incorporating factors from the univariate analyses with p≤0.10. To identify risk factors that independently contribute to sickle cell maculopathy, a stepwise selection procedure was used required a p-value ≤0.10 for a variable to enter the model and a p-value ≤0.10 to remain in the model.

Sickle Cell Disease patients and matched controls were used to compare retinal layers and choroidal thicknesses located central and temporal to the fovea. A univariate analysis of variance model was fit to each retinal layer variable incorporating factors for pair (includes 30 SCD/control matched pairs), eye (OD, OS) and group (SCD, control). These same retinal layer thickness variables were fit, using SCD patients only, to compare patients with sickle cell maculopathy in one or both eyes versus patients without sickle cell maculopathy. A univariate analysis of variance model was fit to each retinal layer variable incorporating factors for patients (30 SCD patients), eye (OD, OS) and sickle cell maculopathy group.

Statistical analyses were performed using SAS statistical software version 9.2 (SAS Inc, Cary, NC). ANOVA models were fit using PROC GLM and logistic regression models fit using PROC LOGISTIC.

## Results

Fifty-nine eyes of 30 consecutive patients with SCD were included in the study. There were 18 women and 12 men. Eighteen patients were Caucasian, 7 African-American, and 5 with Hispanic origin. The mean age of the patients was 38.7 years (range, 22–70 years) and included 9 HbSS (30%), 17 HbS/β° (56.5%), and 4 HbSC (13.5%) (Table 1). Data on both eyes were collected and analyzed, except for 1 eye that was excluded due to anisometropic amblyopia. Best correct visual acuity ranged from 20/25 to 20/20. Due to known allergies, FA was not performed to 2 patients. Across 28 patients who underwent FA, 16 eyes (29%) showed no sign of retinopathy, 23 eyes (42%) showed nonproliferative retinopathy, and 16 eyes (29%) showed proliferative retinopathy. Twelve out of 59 eyes (20%) underwent limited focal laser treatment. Twenty-one out of 55 eyes (38%) showed macular vascular irregularities on FA.

**Table 1:** Baseline characteristics of the sickle cells disease population of this study.

**Age, years**		
	Mean ± SD	38.7 ± 9.89
	Min	22
	Max	70
**Gender, n (%)**		
** **	Females	18 (60%)
** **	Males	12 (40%)
**SCD, n (%)**		
** **	HbSS	9 (30%)
** **	HbS/β^0^	17 (56.5%)
** **	HbSC	4 (13.5%)
**Race, n (%)**		
** **	Caucasian	18
** **	African-American	7
** **	Hispanic	5

SD, standard deviation; SCD, sickle cell disease

Thirteen out of 30 SCD patients (43%), for a total of 19 out of 59 eyes (32%), were noted to have sickle cell maculopathy, detected as patchy areas of severe thinning on SD-OCT thickness maps (Fig 2). The patchy areas of retinal thinning were located temporally to the macula in 19 out of 19 eyes (100%), while 13 out of 19 eyes (68.5%) also showed an involvement of the temporal quadrant of the macula itself. Univariate analysis (Table 2) revealed a significant correlation between the presence of sickle cell maculopathy and transfusion therapy (p = 0.0123), chelation treatment (p = 0.0076), HbF (p = 0.0278), neutrophils (p = 0.0364), acid uric (p = 0.0462), ferritin (p = 0.0346), and transferrin saturation (p = 0.0038). However, the most predictive factors for the occurrence of sickle cell maculopathy as assessed after multivariate regression analysis was chronic chelation treatment (p = 0.0187). HbF level also tended to predict occurrence of this diseases (p = 0.0775) (Table 3). More specifically, the odds of sickle cell maculopathy were 94.2% lower when chelation was present, and the odds decreased by 12.9% when HbF increased by 1%.

**Table 2:** Univariate analysis for sickle cell maculopathy.

Factors	Sickle maculopathy present	Sickle maculopathy absent	p value
Gender (female)	62%	24%	0.0610
Age (y)	39.8	38.7	0.7568
Ethnicity (white)	46%	71%	0.3891
SCD type			
SS	38%	71%	0.2300
SC	38%	24%	
Sb	23%	6%	
Transfusions (Y)	61%	94%	**0.0123**
Chelation (Y)	8%	59%	**0.0076**
Oncocarbide (N)	77%	41%	0.0711
Splenectomy (N)	46%	41%	1.0000
Ischemic events status (N)	62%	53%	0.7273
HbF (%)	5.2	15.0	**0.0278**
HbS (%)	67.3	56.7	0.1321
Reticulocytes (%)	0.225	0.245	0.6652
Leucocytes (%)	7.3	8.8	0.1105
Neutrophils (%)	3.6	5.1	**0.0364**
Hemoglobin (g/dL)	9.9	9.6	0.6379
Hematocrit (%)	28.4	27.9	0.7980
LDH (U/L)	328.1	358.5	0.5492
Urea (mg/dL)	26.3	28.9	0.7319
Uric acid (mg/dL)	5.7	4.6	**0.0462**
Bilirubin-tot (mg/dL)	1.8	2.3	0.1932
Bilirubin-direct (mg/dL)	0.61	0.71	0.2612
AST (U/L)	36.7	42.5	0.6032
ALT (U/L)	26.1	38.4	0.4017
Ferritin (ng/mL)	341.9	1306	**0.0346**
Transferrin saturation (%)	25.0	48.0	**0.0038**
MCV (fL/red cell)	73.5	78.5	0.2210
Platelets (GI/L)	288.4	369.5	0.1958
Creatinine (mg/dL)	0.80	0.72	0.6041

SCD, sickle cell disease; LDH, lactate dehydrogenase; AST, aspartate aminotransferase; ALT, alanine aminotransferase; MCV, mean corpuscular volume

**Table 3:** Results of the stepwise logistic regression model for sickle cell maculopathy.

Odds Ratio Estimates				
Effect	Point Estimate	95% Wald Confidence Limits		Pr > ChiSq
Chelation (N vs Y)	0.058	0.005	0.062	0.0187
HbF	0.871	0.748	1.015	0.0775

While investigating the relationship between severity of sickle cell retinopathy and the presence of sickle cell maculopathy, we found that the prevalence of sickle cell maculopathy was higher in eyes with more severe sickle cell retinopathy; in particular, patchy areas of severe retinal thinning were present in 62.5% (10/16) of the eyes with proliferative retinopathy, 30% (7/23) of the eyes with nonproliferative retinopathy, and 6% (1/16) of the eyes without retinopathy (p = 0.003). Furthermore, the prevalence of sickle cell maculopathy tended to be greater in eyes showing macular vascular abnormalities on FA (47%, 10/21) in comparison to eyes with normal FA (23.5%, 8/34).

**Fig 3:**
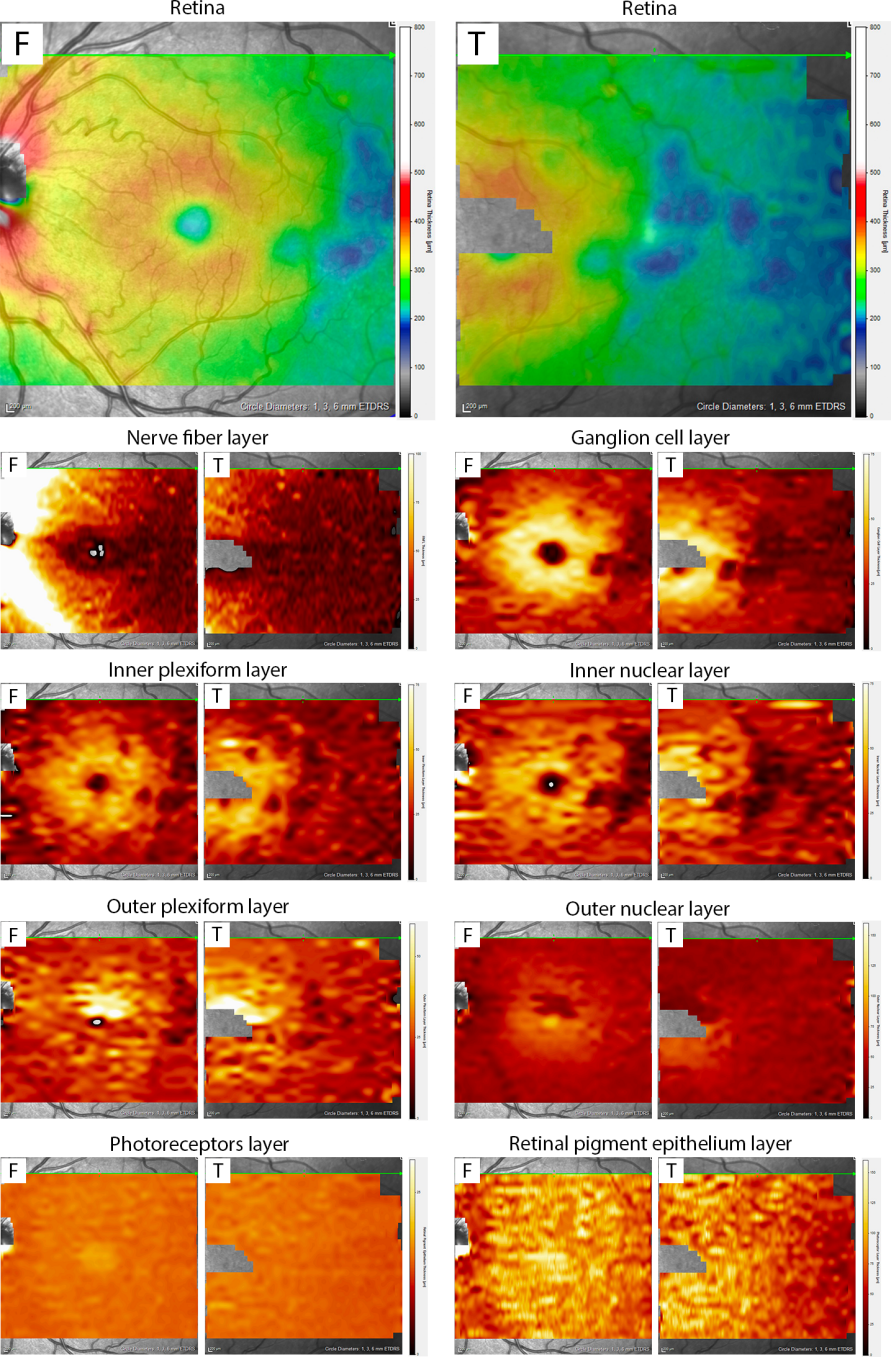
Example of individual retinal layer thickness measurement in subject with sickle cell maculopathy, for both the foveal-centered (“F”) and temporally centered (“T”) scans. Areas of severe thinning are well recognizable as blue patchy areas on retinal thickness color-coded maps (first row), and correspond to darker areas on the thickness maps for ganglion cell layer (second row), inner plexiform layer and inner nuclear layer (third row), and outer plexiform layer (fourth row). The thickness maps for photoreceptors and retinal pigment epithelium do not show any patchy thinning.

Pairwise comparison between SCD patients (with or without sickle cell maculopathy) and normal controls showed that SCD patients had a generalized thinning of the total retinal thickness, both of the macula and of the temporal retina (see Table 4 for details). In particular, most quadrants of the central ETDRS grid showed a significant thinning of multiple retinal layers including GCL, IPL, INL, and ONL (with OPL reduced only inferiorly), while all the quadrants of the temporal ETDRS grid showed a significant thinning of both the INL and ONL (see Table 5 for details). Thickening of the RPE was detected in SCD eyes compared to controls in all quadrants, both centrally and temporally (see Table 5 for details). An example of thickness maps and measurements for each retinal layer from a SCD patient of our cohort can be found in Fig 3. Choroidal volume was similar between SCD eyes and control eyes.

**Table 4:** Comparison of total retinal thickness of the macular region and of the temporal retina between sickle cell disease eyes and control subjects.

* *	ETDRS grid quadrant	Thickness (μm)		
		SCD subjects (n = 59)	Controls (n = 59)	p-value
***Macula***	*Fovea*	267.2	282.7	**<0.0001**
	*Inferior*	312.3	320.4	**0.0014**
	*Nasal*	325.8	337.4	**<0.0001**
	*Superior*	316.6	328.9	**<0.0001**
	*Temporal*	298.7	312.0	**<0.0001**
Temporal Retina*	*Inferior*	232.6	240.9	**0.0017**
	*Superior*	236.3	241.0	**0.0114**
	*Temporal*	215.8	224.0	**0.0006**

SCD, sickle cell disease.

*Thickness data from the nasal quadrant of the temporally-centered grid were not collected because of overlapping with the temporal quadrant of the fovea-centered grid

**Table 5:** Comparison of individual retinal layer thickness of the macular region and of the temporal retina between sickle cell disease eyes and control subjects.

*Retinal layer*	ETDRS grid quadrant	Fovea-centered scan thickness (μm)			Temporally-centered scan thickness (μm)		
		SCD subjects (n = 59)	Controls (n = 59)	p-value	SCD subjects (n = 59)	Controls (n = 59)	p-value
***NFL***	*Inferior*	34.070	34.671	0.3859	19.561	19.543	0.9719
	*Nasal*	37.256	36.561	0.2159	n/a	n/a	n/a
	*Superior*	31.268	31.319	0.9366	18.862	17.888	0.0134
	*Temporal*	18.884	18.163	0.0006	16.126	16.081	0.8901
***GCL***	*Inferior*	42.234	43.615	0.0316	22.925	23.997	0.0692
	*Nasal*	43.950	46.200	0.0004	n/a	n/a	n/a
	*Superior*	42.570	45.205	0.0003	21.934	22.014	0.8759
	*Temporal*	39.377	43.387	<0.0001	17.221	17.176	0.9216
***IPL***	*Inferior*	33.984	35.026	0.0407	21.516	21.846	0.4734
	*Nasal*	35.661	37.161	0.0007	n/a	n/a	n/a
	*Superior*	34.424	36.102	0.0005	21.858	22.823	0.0795
	*Temporal*	35.653	37.772	0.0009	21.858	22.823	0.0795
***INL***	*Inferior*	36.457	37.788	0.0316	24.375	26.599	0.0011
	*Nasal*	37.070	38.308	0.0452	n/a	n/a	n/a
	*Superior*	36.732	37.427	0.2377	24.760	26.180	0.007
	*Temporal*	34.443	36.850	0.0004	21.101	23.235	0.0007
***OPL***	*Inferior*	29.633	32.590	0.0009	23.260	22.938	0.4468
	*Nasal*	30.619	30.449	0.762	n/a	n/a	n/a
	*Superior*	33.146	29.485	0.0001	24.486	23.048	<0.0001
	*Temporal*	29.816	30.113	0.7028	21.410	21.401	0.9831
***ONL***	*Inferior*	56.966	58.135	0.3464	46.056	51.047	<0.0001
	*Nasal*	60.649	67.912	<0.0001	n/a	n/a	n/a
	*Superior*	57.516	68.829	<0.0001	47.523	53.684	<0.0001
	*Temporal*	60.316	66.655	<0.0001	44.907	49.826	<0.0001
***PHOT***	*Inferior*	79.362	78.659	0.117	75.271	74.798	0.2325
	*Nasal*	80.722	80.959	0.6303	n/a	n/a	n/a
	*Superior*	80.870	80.573	0.5748	76.786	75.670	0.0146
	*Temporal*	79.885	79.334	0.2364	74.711	73.729	0.0094
***RPE***	*Inferior*	15.712	14.034	<0.0001	12.662	11.243	<0.0001
	*Nasal*	16.0470	14.928	<0.0001	n/a	n/a	n/a
	*Superior*	16.402	14.953	<0.0001	13.246	11.702	<0.0001
	*Temporal*	15.256	13.553	<0.0001	11.907	10.550	<0.0001

SCD, sickle cell disease; NFL, retinal nerve fiber layer; GCL, ganglion cell layer; IPL, inner plexiform layer; INL, inner nuclear layer; OPL, outer plexiform layer; ONL, outer nuclear layer; PHOT, photoreceptor layer; RPE retinal pigment epithelium; n/a = not applicable (thickness data from the nasal quadrant of the temporally-centered grid were not collected because of overlapping with the temporal quadrant of the fovea-centered grid).

The comparison between SCD eyes with and without sickle cell maculopathy didn’t show any statistical difference in the thickness of any layer in the central macula. However, temporally to the macula it was noted a generalized retinal thinning in the superior and temporal quadrants (see Table 6 for details). More specifically, GCL, IPL, INL, OPL and ONL had a significant thinning in the temporal quadrant (Table 7) with INL and OPL thinned also in the superior and inferior sectors respectively. There was no significant difference in RPE thickness and choroidal volume measurements, neither centrally or temporally (Table 7).

**Table 6:** Comparison of total retinal layer thickness of the macular region and of the temporal retina between sickle cell disease eyes with and without sickle cell maculopathy.

* *	ETDRS grid quadrant	Thickness (μm)		
		Sickle maculopathy absent (n = 40)	Sickle maculopathy present (n = 19)	p-value
***Macula***	*Fovea*	266.5	268.0	0.7514
	*Inferior*	312.4	313.3	0.9097
	*Nasal*	324.6	327.9	0.4390
	*Superior*	315.9	317.7	0.7285
	*Temporal*	299.0	297.5	0.9793
Temporal Retina*	*Inferior*	237.8	224.2	0.0905
	*Superior*	242.2	226.4	**0.0130**
	*Temporal*	225.2	119.9	**<0.0001**

*Thickness data from the nasal quadrant of the temporally-centered grid were not collected because of overlapping with the temporal quadrant of the fovea-centered grid

**Table 7:** Comparison of individual retinal layer thickness of the macular region and of the temporal retina between sickle cell disease eyes with and without sickle cell maculopathy.

*Retinal layer*	ETDRS grid quadrant	Fovea-centered scan thickness (μm)			Temporally-centered scan thickness (μm)		
		Sickle maculopathy absent (n = 40)	Sickle maculopathy present (n = 19)	p-value	Sickle maculopathy absent (n = 40)	Sickle maculopathy present (n = 19)	p-value
***NFL***	*Inferior*	34.445	33.550	0.7421	20.073	18.812	0.5024
	*Nasal*	37.123	37.568	0.7730	n/a	n/a	n/a
	*Superior*	31.754	30.629	0.5540	19.742	17.627	0.1437
	*Temporal*	18.662	19.267	0.4816	16.815	14.940	0.0655
***GCL***	*Inferior*	42.525	41.775	0.7549	24.216	20.738	0.0662
	*Nasal*	43.284	45.080	0.4550	n/a	n/a	n/a
	*Superior*	42.025	43.510	0.4732	23.188	19.943	0.0504
	*Temporal*	40.345	41.765	0.4350	23.332	17.207	0.0020
***IPL***	*Inferior*	34.128	33.723	0.8285	22.060	20.766	0.3723
	*Nasal*	35.636	35.706	0.9616	n/a	n/a	n/a
	*Superior*	34.579	34.204	0.7996	22.678	20.368	0.1484
	*Temporal*	35.349	36.074	0.8098	21.371	18.985	0.0170
***INL***	*Inferior*	35.096	38.711	0.0858	25.433	22.519	0.2247
	*Nasal*	37.014	37.169	0.9234	n/a	n/a	n/a
	*Superior*	36.181	37.611	0.4174	26.472	21.864	0.0184
	*Temporal*	34.787	33.652	0.6003	23.332	17.207	0.0020
***OPL***	*Inferior*	30.919	27.479	0.2391	24.285	21.557	0.0406
	*Nasal*	29.760	32.070	0.2588	n/a	n/a	n/a
	*Superior*	31.490	35.935	0.2120	25.264	23.182	0.1008
	*Temporal*	30.217	29.217	0.6562	23.475	17.904	0.0035
***ONL***	*Inferior*	55.971	58.601	0.4747	46.672	45.128	0.1988
	*Nasal*	61.566	59.046	0.2327	n/a	n/a	n/a
	*Superior*	59.429	54.309	0.2024	48.047	46.650	0.2404
	*Temporal*	59.784	61.039	0.6126	46.189	42.804	0.0207
***PHOT***	*Inferior*	79.126	79.651	0.3798	75.335	75.156	0.7530
	*Nasal*	80.447	81.092	0.4395	n/a	n/a	n/a
	*Superior*	80.650	81.140	0.5266	76.864	76.723	0.8407
	*Temporal*	79.821	79.941	0.8385	74.794	74.723	0.9277
***RPE***	*Inferior*	15.821	15.461	0.4413	12.755	12.560	0.6463
	*Nasal*	16.179	15.804	0.5680	n/a	n/a	n/a
	*Superior*	16.368	16.418	0.9473	13.313	13.215	0.8257
	*Temporal*	15.281	15.201	0.8848	11.845	12.117	0.5798

SCD, sickle cell disease; NFL, retinal nerve fiber layer; GCL, ganglion cell layer; IPL, inner plexiform layer; INL, inner nuclear layer; OPL, outer plexiform layer; ONL, outer nuclear layer; PHOT, photoreceptor layer; RPE retinal pigment epithelium; n/a = not applicable (thickness data from the nasal quadrant of the temporally-centered grid were not collected because of overlapping with the temporal quadrant of the fovea-centered grid).

## Discussion

Systemic risk factors for the occurrence of sickle cell maculopathy have not been previously investigated; however, preventing the occurrence of patchy areas of severe retinal thinning is critical to minimize risk of irreversible visual function loss. Indeed, decreased retinal sensitivity and macular scotoma were demonstrated in eyes with severe macular thinning.[3, 13, 17] An unreported and striking finding from our analysis, chelation therapy was identified as the most protective factor against occurrence of sickle cell maculopathy, potentially followed by HbF levels. The odds of severe retinal thinning was 94.2% lower in case of ongoing chelation therapy, and the odds of severe retinal thinning decreased by 12.9% when HbF increased by 1%. Sickle cells patients have chronic anemia, which is partially corrected by occasional transfusions. Blood transfusions also decrease the percentage of HbS and reduce hemolysis, therefore reduce complications in SCD patients.[18] Indeed, in SCD dense red blood cells containing polymerized HbS may be responsible for repeated reversible arteriolar occlusions resulting in areas of retinal non-perfusion.[19] Therefore, chronic hypoxia may explain the increased risk of retinal thinning in patients not regularly transfused, with iron overload, and that do not receive chronic iron chelation therapy. As retinal and cerebral microvasculatures share many morphological and physiological properties, this hypothesis is also confirmed by previous randomized controlled trials[20], which have demonstrated that regular blood transfusion therapy prevents strokes in children with SCD. However, considering the observational nature of our study, further interventional studies are necessary to confirm our finding. With regard to the potentially protective role of HbF, our results are confirmed by a previous study conducted on 123 children with HbSS.[21] Children with a HbF <15% had higher odds of developing retinopathy; in addition, children treated with hydroxyurea but with retinopathy had lower HbF levels compared to children without retinopathy. This suggests that induction of HbF with hydroxyurea may prevent macular and peripheral ischemia in SCD patients, and therefore potentially reduce the occurrence of sickle cell maculopathy due to capillary occlusion. A relationship between the presence of retinal thinning and SCD subtype (HbSS and HbS/β0) was reported in a previous study[8] but not in the present study. This could be explained by the unequal distribution of sickle cells subtypes and the limited sample size of our cohort.

Sickle cell maculopathy represents a common finding in SCD patients[6–8], as also confirmed by our study where 43% of our SCD population presented with patchy areas of severe retinal thinning on OCT, consistently present temporally to the macula but sometimes also involving the temporal quadrant of the macula itself. The prevalence of sickle cell maculopathy was higher in cases of proliferative retinopathy (62.5%), and when vascular irregularities were detected in the macula on FA (47%). These results confirm the findings from previous studies. Mathew et al observed areas of retinal thinning in the temporal macular area in 44% of the SCD eyes, and PSR was more prevalent in these eyes compared with SCD eyes with normal macular morphology.[8] Brasileiro et al observed focal retinal thinning in 35% of the SCD patients, with a higher frequency in eyes with proliferative changes.[6] In a study of Ghasemi Falavarjani et al[9] inner retinal atrophy was detected in 11 eyes (61.1%) and was associated with a higher ischemic index, which is a parameter previously used to quantify retinal ischemia in patients with retinal vein occlusion.[22] Overall, these findings suggest that macular ischemia and peripheral ischemia are related. Terminal arteriolar branches supply both the temporal macular area and the retinal periphery, and therefore subclinical ischemia due to vascular occlusion may easily occur in these areas in patients with SCD; this may explain the association between the presence of sickle cell maculopathy and the severity of sickle cell retinopathy. The predilection of the temporal macula to focal retinal thinning has been described as *“retinal depression sign”* by Goldbaum in 1978 [23] This finding may be due to the smaller caliber of the end arterioles in the temporal macula compared with that in the nasal region[8], or may be attributable to the fact that temporal macula is a watershed area between the vascular arcades of the retinal circulation.

To better characterize which specific layers are mostly affected in SCD, we measured the thickness of each single retinal layer using an automated segmentation software that was previously validated.[16] To the best of our knowledge, this is the first study that has used single-layer retinal segmentation of OCT scans in SCD patients. Interestingly, we found a generalized thinning of the macular and temporal retina in SCD patients compared to matched controls. Such thinning involved both inner and outer retinal layers (including GCL, IPL, INL, OPL, and ONL centrally; INL and ONL temporally). Moreover, cases with sickle cell maculopathy were affected by an even more pronounced thinning temporally to the macula, involving both inner and outer layers and including the same layers (GCL, IPL, INL, OPL and ONL). Previous OCT-based studies, conducted without automated segmentation, have shown controversial results.[6–8] Both studies conducted by Brasileiro et al and Mathew et al demonstrated inner retinal involvement only.[6, 8] Instead, Hoang et al reported a significant total retinal thinning in the central macula, but with involvement of the outer retinal layers only.[7] The exact etiology of macular thinning in the absence of clinically apparent nonperfusion, however, still remains unclear. The deep capillary plexus is likely to be the most vulnerable retinal plexus to an ischemic insult, as it may reside in a watershed region of oxygen supply.[24] Since most of SCD patients don’t show any acute symptoms, deep retinal plexus ischemia may presumably reflect a chronic loss of the retinal vascular flow. Furthermore, as in our study the retinal thinning was not limited to the layers supplied by the deep capillary plexus, different levels of ischemia could explain the more widespread retinal damage. Indeed, ischemic insults may first involve the deep capillary plexus and consequently further involve the inner retinal circulation. This hypothesis is confirmed by recent OCT-A studies, showing that nonperfusion or reduced flow in both superficial and deep capillary plexi may be responsible for macular thinning in SCD subjects.[10–12]

A new finding of this study is that the retinal thinning involved also the ONL, which receives oxygen and nutrients from the choriocapillaris. Vaso-occlusive events involving the choriocapillaris in SCD patients could explain such ONL thinning but we are unable to prove this due to 3 main reasons. First, the choriocapillaris accounts for the smallest amount of the choroidal thickness[25], and focal infarctions of the choriocapillaris only may not translate into a structural change of the overall choroid. Therefore, measuring the choroid by structural OCT may not be ideal to detect focal infarctions of the choriocapillaris; instead, evaluating the vascular flow using OCT-A technology might better elucidate the involvement of the choriocapillary vasculature in SCD eyes. Second, we measured the choroidal volume since it provides a more comprehensive assessment of the posterior choroid compared to the single-point thickness measurement strategy.[15] However, measuring the overall volume of the posterior choroid does not allow to detect focal choroidal tissue abnormalities, as instead previously described by histological studies reporting spontaneous choroidal vascular occlusions and focal infarctions in patients affected by sickle cell retinopathy.[26, 27] Mathew et al have recently reported a significantly lower choroidal thickness in SCD patients compared to controls by means of OCT.[8] Third, in this study we were not able to control for the multiple factors that have been shown to influence the choroidal volume or thickness, such as eye axial length or time of the OCT scan.[28, 29]

Interestingly, in our study, RPE was consistently found to be thicker in SCD eyes than controls. Although this finding is controversial, it could potentially support the presence of vaso-occlusive disease of the choriocapillaris in SCD patients. Thus, there is a growing evidence that RPE hypertrophy is a cardinal feature of the stress response triggered by various perturbations, such as oxidative damage and iron accumulation. However, only animal studies have been conducted so far; post mortem histological studies are necessary to confirm the potential involvement of RPE as a target tissue for oxidative damage in humans.[30, 31]

This study has a number of limitations. First, the sample size was limited; however, it was sufficient to inform about the primary endpoint of the study as well as to detect meaningful and consistent differences between SCD patients and controls. Second, despite the exclusion of patients with previous panphotocoagulation treatment, we included 12 eyes that had previously undergone focal laser to the periphery. Although unlikely, given that focal laser was localized only in a small portion of the periphery, vascular findings and macular thickness may have been affected by laser treatment in this limited number of eyes. Third, further studies with OCTA are needed to better elucidate the vascular changes in sickle cell maculopathy and to find a correlation with the thinning of specific retinal layers.

In conclusion, we demonstrated for the first time that chelation therapy likely plays a crucial role in the prevention of sickle cell maculopathy, and HbF may also have a protective role. The presence of sickle cell maculopathy is associated with more severe forms of sickle cell retinopathy, confirming a potential correlation between macular and peripheral ischemia. Furthermore, a generalized thinning of the macula is present in SCD patients compared to controls. Thinning involves retinal layers also in eyes without significant signs of macular nonperfusion on FA, suggesting that subclinical ischemia caused by chronic occlusion may happen both in the superficial and deep capillary plexi. The outer retina thinning in SCD patients also suggests that vaso-occlusive ischemic events may occur in the choriocapillaris as well. Because severe retinal thinning affects visual function and can lead to irreversible visual loss, we suggest regular ocular checkups for SCD patients.
